# Maternal Near Miss in Patients with Systemic Lupus Erythematosus

**DOI:** 10.1055/s-0042-1759633

**Published:** 2023-03-06

**Authors:** Arlley Cleverson Belo da Silva, Sue Yazaki Sun, Felipe Favorette Campanharo, Letícia Tiemi Morooka, José Guilherme Cecatti, Rosiane Mattar

**Affiliations:** 1 Escola Paulista de Medicina, Universidade Federal de São Paulo, São Paulo, SP, Brazil; 2 Universidade Estadual de Campinas, Campinas, SP, Brazil

**Keywords:** maternal near miss, severe maternal morbidity, systemic lupus erythematosus, high risk pregnancy, potentially life-threatening conditions, *near miss* materno, morbidade materna grave, lúpus eritematoso sistêmico, gravidez de alto risco, condições potencialmente ameaçadora à vida

## Abstract

**Objective** Systemic lupus erythematosus (SLE) may cause irreversible organ damage. Pregnancy with SLE may have severe life-threatening risks. The present study aimed to determine the prevalence of severe maternal morbidity (SMM) in patients with SLE and analyze the parameters that contributed to cases of greater severity.

M **ethods** This is a cross-sectional retrospective study from analysis of data retrieved from medical records of pregnant women with SLE treated at a University Hospital in Brazil. The pregnant women were divided in a control group without complications, a group with potentially life-threatening conditions (PLTC), and a group with maternal near miss (MNM).

**Results** The maternal near miss rate was 112.9 per 1,000 live births. The majority of PLTC (83.9%) and MNM (92.9%) cases had preterm deliveries with statistically significant increased risk compared with the control group ( *p* = 0.0042; odds ratio [OR]: 12.05; 95% confidence interval [CI]: 1.5–96.6 for the MNM group and *p* = 0.0001; OR: 4.84; 95%CI: 2.2–10.8 for the PLTC group). Severe maternal morbidity increases the risk of longer hospitalization ( *p* < 0.0001; OR: 18.8; 95%CI: 7.0–50.6 and *p* < 0.0001; OR: 158.17; 95%CI: 17.6–1424,2 for the PLTC and MNM groups, respectively), newborns with low birthweight ( *p* = 0.0006; OR: 3.67; 95%CI: 1.7–7.9 and *p* = 0.0009; OR: 17.68; 95%CI: 2–153.6) for the PLTC and MNM groups, respectively] as well as renal diseases (PLTC [8.9%; 33/56; *p* = 0.0069] and MNM [78.6%; 11/14; *p* = 0.0026]). Maternal near miss cases presented increased risk for neonatal death ( *p* = 0.0128; OR: 38.4; 95%CI: 3.3–440.3]), and stillbirth and miscarriage ( *p* = 0.0011; OR: 7.68; 95%CI: 2.2–26.3]).

**Conclusion** Systemic lupus erythematosus was significantly associated with severe maternal morbidity, longer hospitalizations, and increased risk of poor obstetric and neonatal outcomes.

## Resumo

**Objetivo** : Lúpus eritematoso sistêmico (LES) pode causar danos irreversíveis aos órgãos. A gravidez com LES pode ter riscos para condições ameaçadoras à vida. O presente estudo teve como objetivo determinar a prevalência de MMG em pacientes com LES e analisar os parâmetros que contribuíram para os casos de maior gravidade.

**Métodos** Trata-se de um estudo transversal retrospectivo a partir da análise de dados obtidos de prontuários de gestantes com LES atendidas em um Hospital Universitário no Brasil. As gestantes foram divididas em grupo controle sem intercorrências, grupo com condições potencialmente ameaçadoras a vida (CPAV) e grupo com *near miss* materno (NMM).

**Resultados** A taxa de NMM foi de 112,9 por 1.000 nascidos vivos. A maioria dos casos de CPAV (83,9%) e NMM (92,9%) teve partos prematuros com risco aumentado estatisticamente significativo em comparação com o grupo controle ( *p* = 0,0042; odds ratio [OR]: 12,05; intervalo de confiança [IC]: 1,5–96,6 para o grupo NMM e *p* = 0,0001; OR: 4,84; IC95%: 2,2–10,8 para o grupo CPAV). MMG aumenta o risco de maior tempo de internação ( *p* < 0,0001; OR: 18,8; IC95%: 7,0–50,6 e *p* < 0,0001; OR: 158,17; IC95%: 17,6–1424,2 para os grupos CPAV e NMM, respectivamente), recém-nascidos com baixo peso ( *p* = 0,0006; OR: 3,67; IC95%: 1,7–7,9 e *p* = 0,0009; OR: 17,68; IC95%: 2–153,6 para os grupos CPAV e NMM, respectivamente), bem como doenças renais (CPAV: 58,9%; 33/56; *p* = 0,0069 e NMM: 78,6%; 11/14; *p* = 0,0026)]. Os casos de NMM apresentaram risco aumentado para óbito neonatal ( *p* = 0,0128; OR: 38,4; IC95%: 3,3–440,3), natimorto e aborto espontâneo ( *p* = 0,0011; OR: 7,68; IC95%: 2,2–26,3).

**Conclusão** Lúpus eritematoso sistêmico foi significativamente associado à morbidade materna grave, internações mais longas e risco aumentado de desfechos obstétricos e neonatais ruins.

## Introduction

Systemic lupus erythematosus (SLE) is an autoimmune inflammatory disease of multifactorial etiology and involving different systems; thus, it presents diverse clinical manifestations with periods of exacerbations and remissions of signs and symptoms, which may progress to irreversible organic damage. 1 However, there have been improvements in therapeutic strategies over the last decades due to increasing knowledge of the pathophysiology of the disease. 2 3 4

Systemic lupus erythematosus is diagnosed most often in women of child-bearing age. 5 6 Maternal and fetal complications, including miscarriage, fetal death, prematurity, premature rupture of ovular membranes, preeclampsia, acute fetal distress and intrauterine growth restriction can occur during gestation in patients with SLE. 7 Conception is recommended within 6 months of disease remission, as this and other factors, such as absence of chronic hypertension and renal insufficiency, are related to a lower rate of active disease during gestation and a better obstetric outcome. 6 7 8

The maternal mortality ratio is one of the most important indicators in assessing the quality of obstetric practice and perinatal care. Furthermore, researchers worldwide have been studying the prevalence of severe maternal morbidity as an assessment tool in maternal healthcare.

The Maternal Morbidity Working Group of the World Health Organization (WHO) defined uniform criteria aiming to identify cases of severe maternal morbidity (SMM), potentially life-threatening conditions (PLTCs) and maternal near miss (MNM). 9

The maternal near miss case is known as a woman who nearly died but survived a severe complication that occurred during pregnancy, childbirth, or within 42 days of termination of pregnancy. Moreover, identification of cases with some PLTCs would be useful for prospective surveillance on severe complications among which maternal near miss cases would emerge. 9

Charts 1 and 2 present the list of life-threatening conditions proposed as the criteria for identification of maternal near miss cases and potentially life-threatening conditions cases. 9

**Chart 1:** The WHO maternal near miss criteria: a woman presenting with any of the following life-threatening conditions and surviving a complication that occurred during pregnancy, childbirth or within 42 days of termination of pregnancy should be considered as a maternal near miss case

Clinical criteria	
Acute cyanosis	Loss of consciousness lasting 12 hours ^e^
Gasping a	Loss of consciousness AND absence of pulse/heartbeat
Respiratory rate > 40 or < 6/min	Stroke ^f^
Shock ^b^	Uncontrollable fit/total paralysis ^g^
Oliguria nonresponsive to fluids or diuretics ^c^	Jaundice in the presence of pre-eclampsia ^h^
Clotting failure ^d^	
**Laboratory-based criteria**	
Oxygen saturation < 90% for ≥ 60 minutes	pH < 7.1
PaO2/FiO2 < 200mmHg	Lactate > 5
Creatinine ≥ 300 mmol/l or ≥ 3,5 mg/dl	Acute thrombocytopenia (< 50,000 platelets)
Bilirubin >100 µmol/l or > 6,0 mg/dl	Loss of consciousness AND the presence of glucose and ketoacids in urine
**Management-based criteria**	
Use of continuous vasoactive drugs ^i^	Intubation and ventilation for 60 minutes not related to anesthesia
Hysterectomy following infection or hemorrhage	Dialysis for acute renal failure
Transfusion of ≥ 5 units red cell transfusion	Cardiopulmonary resuscitation (CPR)

a Gasping is a terminal respiratory pattern and the breath is convulsively and audibly caught. ^b^ Shock is a persistent severe hypotension, defined as a systolic blood pressure < 90 mmHg for ≥ 60 minutes with a pulse rate at least 120 despite aggressive fluid replacement (> 2l). ^c^ Oliguria is defined as a urinary output < 30 ml/hr for 4 hours or < 400 ml/24 hr. ^d^ Clotting failure can be assessed by the bedside clotting test or absence of clotting from the IV site after 7–10 minutes. ^e^ Loss of consciousness is a profound alteration of mental state that involves complete or near-complete lack of responsiveness to external stimuli. It is defined as a Coma Glasgow Scale < 10 (moderate or severe coma). ^f^ Stroke is a neurological deficit of cerebrovascular cause that persists beyond 24 hours or is interrupted by death within 24 hours. ^g^ Condition in which the brain is in a state of continuous seizure. ^h^ Pre-eclampsia is defined as the presence of hypertension associated with proteinuria. Hypertension is defined as a blood pressure of at least 140 mmHg (systolic) or at least 90 mmHg (diastolic) on at least two occasions and at least 4–6 h apart after the 20 ^th^ week of gestation in women known to be normotensive beforehand. Proteinuria is defined as excretion of 300 mg or more of protein every 24 hours. If 24-hour urine samples are not available, proteinuria is defined as a protein concentration of 300 mg/l or more (≥1 + on dipstick) in at least 2 random urine samples taken at least 4–6 h apart. ^I^ For instance, continuous use of any dose of dopamine, epinephrine, or norepinephrine.

**Chart 2:** Potentially life-threatening conditions

Hemorrhagic disorders	Hypertensive disorders
Abruptio placenta	Severe pre-eclampsia
Accreta/increta/percreta placenta	Eclampsia
Ectopic pregnancy	Severe hypertension
Postpartum hemorrhage	Hypertensive encephalopathy
Ruptured uterus	HELLP syndrome
**Other systemic disorders**	**Severe management indicators**
Endometritis	Blood transfusion
Pulmonary edema	Central venous access
Respiratory failure	Hysterectomy
Seizures	ICU admission
Sepsis	Prolonged hospital stay (> 7 postpartum days)
Shock	Non anesthetic intubation
Thrombocytopenia < 100,000	Return to operating room
Thyroid crisis	Surgical intervention

The main purpose of the present study was to evaluate the association of SLE with maternal near miss, PLTCs and maternal mortality. Although several studies have reported on the link between SLE and maternal morbidity, to the best of our knowledge there are no studies specific to pregnant patients with SLE and SMM, according to the WHO criteria. 9 The secondary aim was to compare sociodemographic, obstetric, perinatal characteristics, and maternal morbidity features between patients.

## Methods

This was a cross-sectional, retrospective study of pregnant patients with systematic lupus erythematosus cared to from January 2005 to December 2015 in the prenatal care unit and labor ward of the Hospital São Paulo from the Universidade Federal de São Paulo – Escola Paulista de Medicina, a tertiary referral service in Brazil. Data collection, held between 2017 and 2018, was conducted separately by two researchers in order to check the consistency of data extracted from written medical records of pregnant women with SLE and their newborns.

Pregnant patients with SLE were divided into three groups: without complications (control group: CG), with PLTCs and patients with MNM, according to the WHO criteria. 9 Study variables included sociodemographic features, clinical and obstetric history, prenatal care, mode of delivery, gestational age at childbirth, days of hospitalization, and obstetric outcome. Birth conditions (Apgar score at 5 minutes and birthweight) and neonatal outcome were included.

The collected data were analyzed using IBM SPSS Statistics for Windows version 23.0 (IBM Corp., Armonk, NY, USA). Descriptive analysis was performed using frequency and percentage for the categorical variables; for continuous variables, mean, standard deviation (SD), minimum, median, and maximum were used. The chi-squared test, the Fisher exact test, and the Likelihood ratio test were used to compare the variables. P-values < 0.05 were considered statistically significant, and odds ratios (ORs) were estimated with their 95% confidence interval (CI).

The study was approved by the Ethics Review Board of Universidade Federal de São Paulo, under CAAE number 56744616.0.0000.5505. The need for informed consent was waived due to the retrospective design of the study. However, all of the authors signed a document guaranteeing the confidentiality and secrecy of data in order to preserve the anonymity of patients.

## Results

**Fig. 1:**
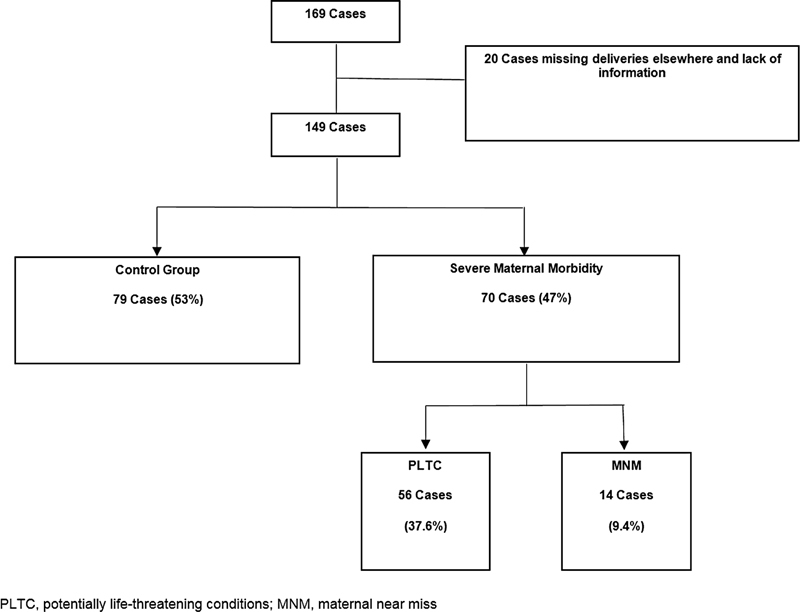
Flow chart of inclusions.

The initial sample consisted of 169 pregnant patients with SLE. Twenty cases were excluded, 6 because childbirth did not occur at the Hospital São Paulo and 14 due to unavailable records. The final 149 cases were divided into 3 groups: control group (CG) ( *n* = 79; 53%); PLTC ( *n* = 56; 37.6%); and MNM ( *n* = 14; 9.4%) ( Fig. 1 ).

The mean age of the participants was 29 years old, with most patient ages ranging between 26 and 35 years old (51%; 76/149 pregnancies). The maternal near miss rate was 112.9 per 1,000 live births; there were no cases of maternal death. There were no statistically significant differences in sociodemographic features or obstetric characteristics (number of pregnancies, parity, and number of prenatal visits) among the three groups ( Table 1 ).

**Table 1:** Sociodemographic and obstetric characteristics between groups of complications of pregnancies with systemic lupus erythematosus

Characteristics	Group			*p-value*	*p-value*
	PLTC	MNM	Control Group	Control Group PLTC	Control Group MNM
Age (years old)					
15 to 25	16 (28.6%)	9 (64.3%)	25 (31,6%)	0.2335 ^c^	0.0647 a
26 to 35	34 (60.7%)	4 (28.6%)	38 (48,1%)		
> 35	6 (10.7%)	1 (7.1%)	16 (20,3%)		
( *n* )	56	14	79		
Ethnic origin ^d^					
White	34 (66.7%)	9 (75%)	37 (53.6%)	0.1507 ^c^	0.1677 ^c^
Other	17 (33.3%)	3 (25%)	32 (46.4%)		
( *n* )	51	12	69		
Education ^e^					
None	6 (11.3%)	2 (18.2%)	9 (15%)	0.9241 ^c^	0.9121 a
Primary school	14 (26.4%)	2 (18.2%)	17 (28.3%)		
High school	28 (52.8%)	6 (54.5%)	29 (48.3%)		
University	5 (9.4%)	1 (9.1%)	5 (8.3%)		
( *n* )	53	11	60		
Marital status ^f^					
With partner	42 (79.2%)	9 (69.2%)	50 (69.4%)	0.2193 ^c^	1.0000 ^b^
No partner	11 (20.8%)	4 (30.8%)	22 (30.6%)		
( *n* )	53	13	72		
Number of pregnancies					
1	25 (44.6%)	6 (42.9%)	24 (30.4%)	0.0895 ^c^	0.3679 ^b^
≥ 2	31 (55.4%)	8 (57.1%)	55 (69.6%)		
( *n* )	56	14	79		
Parity					
0	29 (51.8%)	6 (42.9%)	31 (39.2%)	0.1484 ^c^	0.7989 ^c^
≥ 1	27 (48.2%)	8 (57.1%)	48 (60.8%)		
( *n* )	56	14	79		
Prenatal visits					
0	8 (14.3%)	6 (42.9%)	15 (19%)	0.1638 a	0.0549 a
1–5	12 (21.4%)	4 (28.6%)	14 (17.7%)		
6–12	25 (44.6%)	4 (28.6%)	41 (51.9%)		
13–18	8 (14.3%)	0 (0%)	9 (11.4%)		
> 18	3 (5.4%)	0 (0%)	0 (0%)		
( *n* )	56	14	79		

Abbreviations: MNM, maternal near miss; PLTC, potentially life-threatening condition.

Statistically significant when *p* < 0.05.

a Likelihood Ratio Test; ^b^ Fisher Exact test; ^c^ X ^2^ test adjusted for cluster effect; ^d^ Missing values of 17 women; ^e^ Missing values of 25 women; ^f^ Missing values of 11 women; ^g^ Ectopic pregnancy case excluded from the statistical analysis.

The MNM criteria most frequently identified were “creatinine ≥ 3.5 mg/dl” and “dialysis for acute renal failure”, with at least 1 of these criteria present in half of the cases ( Table 2 ). Descriptive analysis revealed that the PLTC criteria “Prolonged hospital stay” was the most identified within all cases of SMM, both the MNM (85.7%; 12/14) and PLTC (62.6%; 35/56) groups. The duration of hospital stay during pregnancy, delivery, and postpartum was > 8 days in the PLTC (60.7%; 34/56) and MNM (92.9%; 13/14) groups, significantly greater than the CG (7.6%; 6/79) ( *p* < 0.0001; OR: 18.8; 95%CI: 7.0–50.6 and *p* < 0.0001; OR: 158.17; 95%CI: 17.6–1,424.2) for PLTC and MNM, respectively) ( Tables 3 and 4 ). In the MNM group, the length of hospitalization was > 21 days in over half of the patients. In addition, the second most frequent PLTC criteria, within all cases of SMM, was hypertensive complications such as severe hypertension, severe preeclampsia and HELLP syndrome, identified in 55.4% (31/56) and 64.3% (9/14) of the PLTC and MNM groups, respectively.

**Table 2:** Description of the criteria present in each maternal near miss case according to WHO criteria

Patient	Age (years old)	Criteria		
		Clinical	Laboratory	Management
Case 1	31	−	−	Hysterectomy following infection or hemorrhage
Case 2	28	Shock	−	Transfusion of ≥ 5 units of red cells
Case 3	25	Clotting failure	Lactate > 5 / Platelets < 50,000	Transfusion of ≥ 5 units of red cells
Case 4	20	−	−	Transfusion of ≥ 5 units of red cells
Case 5	24	Respiratory rate > 40 or < 6/min	Lactate > 5	−
Case 6	24	−	Oxygen saturation < 90% for ≥ 60 minutes / Lactate > 5	Intubation/ventilation for ≥ 60 min not related to anesthesia
Case 7	22	−	Creatinine ≥ 3.5mg/dl	Dialysis for acute renal failure
Case 8	32	−	Creatinine ≥ 3.5mg/dl	Dialysis for acute renal failure
Case 9	37	−	Creatinine ≥ 3.5mg/dl	Dialysis for acute renal failure
Case 10	25	−	Creatinine ≥ 3.5mg/dl	Dialysis for acute renal failure
Case 11	27	−	Creatinine ≥ 3.5mg/dl	−
Case 12	19	−	−	Dialysis for acute renal failure
Case 13	22	−	Creatinine ≥ 3.5mg/dl / Lactate > 5	−
Case 14	22	−	Lactate > 5	−

**Table 3:** Hospitalization time, obstetric and neonatal outcomes between the PLTC and control groups of pregnancies with systemic lupus erythematosus

Characteristics	Group		*p-value*	OR (95% CI)	
	PLTC	Control Group			
Hospital Stay (days)			< 0.0001	18.8 [7.0–50.6]
< 8 (ref)	22 (39.3%)	73 (92.4%)		
≥ 8	34 (60.7%)	6 (7.6%)		
( *n* )	56	79		
GA at delivery (weeks)			0.0001	4.84 [2.2–10.8]
< 37	47 (83.9%)	41 (51.9%)		
≥ 37 (ref)	9 (16.1%)	38 (48.1%)		
( *n* )	56	79		
Outcome/Mode of delivery				
Miscarriage	1 (1.9%)	12 (15.2%)	0.1301 a	5.78 [0.7–49.3]
Cesarean section	41 (74.5%)	40 (50.6%)	0.0774	0.47 [0.2–1.0]
Vaginal (ref)	13 (23.6%)	27 (34.2%)		−
( *n* )	55 ^b^	79		
Neonatal outcome				
Neonatal death < 7 days	4 (7.5%)	0 (0%)	0.0975 a	12.84 [0.2–57.7]
Neonatal death ≥ 7 days	2 (3.8%)	0 (0%)	0.5275 a	7.13 [0.1–25843.3]
Miscarriage/stillbirth	3 (5.7%)	14 (18.2%)	0.1916	0.31 [0.083–1.1]
Hospital discharge (ref)	44 (83%)	63 (81.8%)		−
( *n* )	53	77		
Birthweight (grams)				
≤ 2,500	32 (59.3%)	19 (28.4%)	0.0006	3.67 [1.7–7.9]
> 2,500	22 (40.7%)	48 (71.6%)		
( *n* )	54 ^c^	67 ^d^		

Abbreviations: GA, Gestational Age; PLTC, potentially life-threatening condition.

Statistically significant when *p* < 0.05.

a Fisher Exact test ^b^ Ectopic pregnancy case excluded from the statistical analysis. ^c^ Missing values of 1 case of miscarriage. ^d^ Missing values of 12 cases of miscarriage.

**Table 4:** Hospitalization time, obstetric and neonatal outcomes between the MNM and control groups of pregnancies with systemic lupus erythematosus

Characteristics	Group		*p-value*	OR (95% CI)
	MNM	Control Group		
Hospital Stay (days)			< 0.0001	158.17 [17.6– 1424.2]
< 8 (ref)	1 (7.1%)	73 (92.4%)		
≥ 8	13 (92.9%)	6 (7.6%)		
( *n* )	14	79		
GA at delivery (weeks)			0.0042	12.05 [1.5–96.6]
< 37	13 (92.9%)	41 (51.9%)		
≥ 37 (ref)	1 (7.1%)	38 (48.1%)		
( *n* )	14	79		
Outcome/mode of delivery				
Miscarriage	6 (42.9%)	12 (15.2%)	0.0899 ^a^	0.22 [0.05–1.04]
Cesarean section	5 (35.7%)	40 (50.6%)	0.5687	0.89 [0.20–4.0]
Vaginal (ref)	3 (21.4%)	27 (34.2%)		−
( *n* )	14	79		
Neonatal outcome				
Neonatal death ≥ 7 days	2 (14.3%)	0 (0%)	0.0128 ^a^	38.4 [3.3–440.3]
Miscarriage/stillbirth	8 (57.1%)	14 (18.2%)	0.0011	7,68 [2.2–26.3]
Hospital discharge (ref)	4 (28.6%)	63 (81.8%)		
( *n* )	14	77		
Birthweight (grams)				
≤ 2,500	7 (87.5%)	19 (28.4%)	0.0009	17,68 [2–153.6]
> 2,500	1 (12.5%)	48 (71.6%)		
( *n* )	8 ^b^	67 ^c^		

Abbreviations: GA, Gestational Age; MNM, maternal near miss.

Statistically significant when *p* < 0.05. ^a^ Fisher Exact test. ^c^ Missing values of 6 cases of miscarriage. ^d^ Missing values of 12 cases of miscarriage.

Delivery after 37 weeks occurred in 16.1 and 7.1% of the PLTC and MNM groups compared with 48.1% of the CG. Consequently, there was increased risk of preterm delivery both in the MNM group ( *p* = 0.0042; OR: 12.05; 95%CI: 1.5–96.6) and PLTC group ( *p* = 0.0001; OR: 4.84; 95%CI: 2.2–10.8) ( Tables 1 and 2 ). Additional analysis demonstrated that 71.5% (10/14) of the MNM group delivered before 27 weeks compared with 16.4% in the CG ( *p* < 0.001) and 70% (39/56) of the PLTC group delivered between 28 and 37 weeks compared to 35.4% (28/79) in the CG (p < 0.001) ( Tables 3 and 4 ).

A total of 57.1% of the MNM group (8/14) resulted in stillbirth and miscarriage compared with 18.2% (14/77) in the CG. Therefore, there is increased risk of pregnancy loss in the MNM group ( *p* = 0.0011; OR: 7.68; 95%CI: 2.2–26.3) ( Table 5 ). Moreover, there is a higher chance of late neonatal death than neonatal hospital discharge in MNM cases ( *p* = 0.0128; OR: 38.4; 95%CI: 3.3–440.3) ( Table 5 ). On the other hand, despite the four cases of early neonatal death in the PLTC group, there was no significantly increased risk of neither neonatal death nor miscarriage/stillbirth when compared with the neonatal hospital discharge rate. Additional analysis demonstrated that newborns from the PLTC group weighed < 1,500 g in 28% (15/54) of the cases compared with 6% (4/67) in the CG ( *p* = 0.0016); half of the MNM newborns (4/8) weighed < 1,000 g, compared to 1.5% (1/67) in the CG ( *p* = 0.0003). Considering the characteristics of low birthweight newborns, there was increased risk in both the PLTC group ( *p* = 0.0006; OR: 3.67; 95%CI: 1.7–7.9) ( Table 1 ) and the MNM group ( *p* = 0.0009; OR): 17.68; 95%CI: 2–153.6). The presence of systemic impairment and antibodies due to SLE were assessed with statistically significant results. Renal disorder had higher prevalence in the PLTC (58.9%; 33/56; *p* = 0.0069) and MNM (78.6%; 11/14; *p* = 0.0026) groups than in the CG (35.4%; 28/79). In addition, hematological disorders had higher prevalence in the MNM group (71.4%; 10/14) compared with the CG (40.5%; 32/79; *p* = 0.032). There was a lower percentage of cutaneous impairment in the MNM group (64.3%; 9/14) compared with the CG (82.3%; 65/79) ( Table 5 ).

**Table 5:** Systemic impairment and positive antibodies between group of complications of pregnancies with systemic lupus erythematosus

	Group			*p-value*	*p-value*
Systemic impairment	PLTC	MNM	Control Group	Control Group PLTC	Control Group MNM
	n =56	n =14	n =79		
Renal disorder	33 (58.9%)	11 (78.6%)	28 (35.4%)	0.0069	0.0026
Cutaneous disorder	36 (64.3%)	9 (64.3%)	65 (82.3%)	0.0177	0.1521 ^a^
Arthritis	39 (69.6%)	9 (64.3%)	57 (72.2%)	0.7513	0.5385 ^a^
Serositis	11 (19.6%)	2 (14.3%)	13 (16.5%)	0.6332	1.0000 ^a^
Blood disorder	28 (50%)	10 (71.4%)	32 (40.5%)	0.2741	0.0321
Neurologic disorder	4 (7.1%)	3 (21.4%)	8 (10.1%)	0.7606 ^a^	0.3619 ^a^
**Antibodies**					
Anti DNA	19 (33.9%)	3 (21.4%)	21 (26.6%)	0.3571	1.0000 ^a^
Anti Smith (Sm)	10 (17.9%)	2 (14.3%)	15 (19%)	0.8677	1.0000 ^a^
Anticardiolipin	11 (19.6%)	1 (7.1%)	6 (7.6%)	0.0376	1.0000 ^a^
Lupus anticoagulant	4 (7.1%)	1 (7.1%)	0 (0%)	0.0278 ^a^	0.1505 ^a^
AntiRo	15 (26.8%)	2 (14.3%)	27 (34.2%)	0.3607	0.2120 ^a^
Anti La	4 (7.1%)	0 (0%)	5 (6.3%)	1.0000 ^a^	1.0000 ^a^
Antinuclear	47 (83.9%)	11 (78.6%)	62 (78.5%)	0.4291	1.0000 ^a^
Anti RNP	14 (25%)	3 (21.4%)	20 (25.3%)	0.9667	1.0000 ^a^

Abbreviations: MNM, maternal near miss; PLTC, potentially life-threatening condition.

Statistically significant when *p* < 0.05. aFisher Exact test.

## Discussion

A relationship between SLE and pregnancy complications has been previously established. The purpose of the present study was to evaluate the association of SLE with SMM according to the WHO standardized definitions and criteria, 9 as well as to compare sociodemographic, obstetric, perinatal characteristics, and maternal morbidity features in this cohort.

The elevated incidence rate of MNM in the present study (112.9 cases per 1,000 live births) demonstrates that SLE has as a severe impact on pregnancy. The range of MNM incidence rates (per 1,000 live births) in the general population according to the WHO criteria is 9.35 to 13.5 in Brazil and Latin American countries, 10 11 12 13 14 15 5.9 in the United States of America, 16 and 21.5 in Rwanda. 17 Additionally, the PLTC and MNM groups revealed a higher frequency of complications, such as prematurity, stillbirth, miscarriage, neonatal death, low birthweight, and a longer period of hospitalization.

We also identified a high association between SMM and maternal and fetal complications, including low birthweight, neonatal death, fetal loss, and miscarriage, correlating with previous reports. 7 18 19 20 21 22 23 Prematurity was one of the most significant complications, with the majority of pregnancies in the MNM group delivering earlier than 27 weeks (71.5%), with statistically significant increased risk of preterm delivery in both the MNM and PLTC groups.

The patients had a statistically significant longer hospitalization period, including "prolonged hospital stay (> 7 days)" as the main criteria in both SMM groups. Therefore, we emphasize the importance of applying strategies that improve the healthcare of these patients through better prenatal follow-up. Such strategies may lower the risk of complications, decreasing long-term hospitalization, and reducing social and economic costs, which benefit public national health systems. As previously mentioned, among the 70 patients with SMM, 56 presented with PLTC and 14 with MNM. Thus, it could be argued that, theoretically, 80% of the PLTC cases were identified and reversed before evolving to MNM cases. This analysis directly reflects service assistance quality and can be useful in audits as well as in subsequent implementations of improvements in health care. 24

The association of renal impairment and SMM among the patients indicated that the presence of lupus nephritis during gestation is a relevant factor for developing complications. Cases with positive antiphospholipid antibodies or antiphospholipid antibody syndrome associated with SLE also had a higher risk of developing PLTC. Several studies have indicated the presence of previous lupus nephritis, chronic hypertension, and positive antiphospholipid antibodies, such as anticardiolipin antibody and lupus anticoagulant, to be predictors of poor obstetric and neonatal outcomes in patients with SLE. 7 8 18 19 20 25 26 27

There were two unexpected results in the present study. One was the relation between hematological impairment and a higher percentage of MNM compared with the CG. The other one was the association of cutaneous criteria (malar rash, discoid rash, and photosensitivity) with a lower percentage of cases with SMM. Such relationships have not been found in previous studies regarding lupus during pregnancy. In order to understand the first finding, the 10 MNM cases with hematological criteria were analyzed individually and it was found that in 7 of them there was also nephritis, with the presence of laboratory and management criteria "creatinine ≥ 3,5 mg/dl " and "dialysis for acute renal failure ", respectively. Therefore, nephritis was believed to be a bias factor for this result. No bias factor was identified for the second unexpected finding; therefore, cutaneous criteria in SLE patients might be considered a weak sign for SMM and poor obstetric outcomes. However, new studies should be performed to define this relationship more clearly.

The present retrospective study has some limitations. Since it was based on existing medical records, some data was missing. Additionally, it might be considered as a limitation the fact of performing retrospective data analysis. Although we selected a 10-year period for the present study to provide an adequate number of patients, in the future, a larger sample size and the collection of prospective data may provide more representative results.

## Conclusion

The findings of the present study demonstrated a strong and significant association between SLE and SMM. Cases of SMM had longer hospitalization and a higher risk of poor obstetric and neonatal outcomes, such as prematurity, miscarriage, fetal loss, and neonatal death compared with the CG. The presence of chronic or acute renal impairment was a significant factor in the evolution of SMM. We believe that the development of SMM in this population might be avoided through appropriate preconception planning, good disease control, absence of disease activity (especially of the renal system), along with specialized prenatal, perinatal, and postpartum care conducted by a multiprofessional team.
